# A paradigm shift in nutritional clinical practice: filling a gap on the implementation of the Dietary Guidelines for the Brazilian Population

**DOI:** 10.20945/2359-4292-2024-0446

**Published:** 2025-01-27

**Authors:** Kamila Tiemann Gabe, Carlos Augusto Monteiro

**Affiliations:** 1 Faculdade de Saúde Pública de São Paulo, Universidade de São Paulo, São Paulo, SP, Brasil

The pursuit of understanding the role of diet in the global epidemic of obesity and chronic diseases has transformed nutrition science over the past decades. Studies focusing on isolated nutrients, such as fats or sugars, proved inadequate in explaining the emerging epidemiological landscape (1). This reductionist perspective was overcome by a broader focus on dietary patterns, initially supported by large clinical trials that demonstrated the protective effects of the Mediterranean diet in reducing the risk of several diseases (2). A major advancement came when researchers from the Center for Epidemiological Research in Nutrition and Health at the University of São Paulo (NUPENS/USP) proposed a novel food classification system, based on the extent and purpose of industrial food processing, opening ways for a new perspective on dietary patterns studies (3). Today, there is solid evidence that the intake of ultra-processed foods, one of the categories in this classification, is a significant driver of the global epidemic of obesity and obesity-related chronic diseases (4).

In Brazil, this evidence has been translated into public policy through the Dietary Guidelines for the Brazilian Population (hereinafter called Guide), a document developed by the Ministry of Health that provides dietary recommendations tailored to the Brazilian population (5). The Guide, now in its tenth year, emphasizes the message “always prefer natural and minimally processed foods and their culinary preparations over ultra-processed foods”, encouraging the traditional Brazilian diet, rich in staples like rice and beans, to form the foundation of our diet. Moreover, the Guide moves away from recommendations based on quantities and portions, operating on the understanding that, except in specific cases, our natural mechanisms of hunger and satiety are sufficient to regulate food intake once ultra-processed foods, which are designed to be consumed in excess, are excluded.

This innovative concept of healthy eating in Brazil prompted the need to rethink nutritional care practices, particularly within the Unified Health System (SUS). In this context, a key challenge was implementing the new recommendations in individualized dietary counseling, as diet planning and advice have historically focused on adjusting macroand micronutrient intake - a practice now outdated by the food processing based dietary pattern studies. The manuscript “Translating the Brazilian Dietary Guidelines into clinical practice: innovative strategies for healthcare professionals”, authored by Vanessa Del Castillo Silva Couto, Patrícia Constante Jaime, and Maria Laura da Costa Louzada, proposes an innovative way to address this gap. The authors describe two tools for incorporating the Guide into the clinical practice of healthcare professionals, including both nutritionists and non-nutritionists (6).

The first tool (Protocols Based on the Brazilian Dietary Guidelines for Individual Dietary Advice) consists of a set of protocols for individualized dietary counseling, which can be used by any healthcare professional and is aimed at individuals with less complex dietary and nutritional needs. Organized into five booklets, each corresponding to a different stage of life, the protocols present flowcharts of recommendations previously described and already made available by the Ministry of Health (7). Prioritized recommendations in this flowchart are those that are more structural to the dietary pattern, such as beans and legumes intake, moving toward more specific ones, focused on enhancing diet quality, such as vegetables intake.

The second tool (Dietary Guidelines-based Meal Plans) involves the development of a personalized meal plan based on the Guide’s recommendations, suitable for more complex cases that require detailed dietary prescriptions. Unlike conventional dietary plan that focus on nutrient profiles, this approach prioritizes the overall eating pattern, emphasizing food processing characteristics. The innovative underlying assumption is that a dietary pattern based on natural and minimally processed foods, with diversity within and between food groups, can meet individual macroand micronutrient needs without requiring detailed nutritional calculations. However, such analysis can be performed as a second step for adjustments in cases where it is deemed necessary (*e.g.*, potassium intake in cases of chronic kidney disease).

Another key aspect of the manuscript by Silva Couto et al is its recognition that effective clinical practice requires understanding of the social determinants of health. Aligned with the Guide principles, this considers that dietary advice should be sensitive to individuals’ life contexts and goals, considering people’s eating habits, social and demographic conditions, living environments, education and workplace contexts, functional capacities, preferences, habits, and culture. This is clearly illustrated in the manuscript through a clinical case study, where the proposed methodologies are applied using an interdisciplinary and holistic approach to address the dietary needs of Theresa.

The work represents a critical advance for the public health in Brazil by filling a practical gap within the SUS. While implementing these new strategies may present challenges, they are undoubtedly simpler than traditional dietary calculations, which could facilitate their adoption. Another significant contribution of the proposal for the SUS is the integration of the Sisvan’s Food Consumption Markers Questionnaire. In this sense, these strategies have the potential to enhance food and nutritional surveillance in Brazil, as their implementation could lead to increased food intake data collection and registration.

Moreover, it also represents a significant advance in the field of implementation science, as it proposes concrete ways for systematically integrating evidence-based dietary recommendations into clinical practice. In this sense, the study raises new research questions, such as understanding the barriers and facilitators for healthcare professionals’ adherence to the proposed methods, as well as their effectiveness in promoting dietary behavior change. Additionally, the study opens pathways for community-level intervention research through randomized clinical trials, which are still scarce in the literature on ultra-processed foods. The proposed methods could be used in studies aiming to investigate whether increased adherence to the Brazilian dietary pattern recommended by the Guide provides a protective effect on health, similar to the large studies conducted on the Mediterranean diet.

In summary, the manuscript presents an innovative proposal aligned with the recent paradigm shift in the field of nutrition. It has the potential to radically change the way nutritionists and other healthcare professionals approach individualized dietary counseling, moving beyond the nutrient-centered perspective that has already been left behind by current research. As such, the manuscript offers a valuable contribution to the nutritional science and public health in Brazil.
